# Clinical and Endoscopic Features of Pneumatosis Cystoides Intestinalis: A Retrospective Study in 192 Patients

**DOI:** 10.5152/tjg.2023.22689

**Published:** 2023-11-01

**Authors:** Yan Wang, Bo Zhang, Longsong Li, Hongyi Sun, Ningli Chai, Enqiang Linghu

**Affiliations:** 1 Nankai University School of Medicine, Tianjin, China; 2 Department of Gastroenterology, The First Medical Center of PLA General Hospital, Beijing, China

**Keywords:** Pneumatosis cystoides intestinalis, colonoscopy, endoscopy, endoscopic ultrasonography

## Abstract

**Background/Aims:**

Pneumatosis cystoides intestinalis is not well recognized. Clinical features vary in several case reports, and prognosis remains unclear. We aimed to summarize the clinical and endoscopic features of pneumatosis cystoides intestinalis and to explore potential factors associated with lesion size.

**Materials and Methods:**

We retrospectively collected clinical and endoscopic features of patients diagnosed with pneumatosis cystoides intestinalis from July 2015 to October 2021. Patients were allocated to 2 groups according to lesion size with 2 cm as boundary value. Baseline characteristics were compared between the groups.

**Results:**

A total of 192 patients were included in this study with a 1.3:1 male-to-female ratio. About 91 lesions (47.70%) were ≥2 cm and those patients were more likely to have a history of polypectomy or abdominal surgery compared to lesion size <2 cm (*P *< .05). For 50 patients who received follow-up colonoscopy, 28 cases (56.00%) disappeared spontaneously and 22 cases (44.00%) remained unchanged. No factors have been observed to be connected with prognosis.

**Conclusion:**

Colonoscopy is beneficial to the diagnosis of pneumatosis cystoides intestinalis. Patients with a history of polypectomy or abdominal surgery were more likely to develop lesions <2 cm. Most patients do not need special treatments and have favorable prognosis.

**Main Points**

- We conducted a maximum sample size study on colonic pneumatosis cystoides intestinalis, a disease that has not been well recognized.
- We explored potential factors to be associated with lesion size and prognosis and found that patients with a history of polypectomy or abdominal surgery were more likely to develop larger lesions, which may lead to more obvious symptoms.
- We systematically summarized clinical and endoscopic features of pneumatosis cystoides intestinalis and were able to improve cognitions toward this disease.

## Introduction

Pneumatosis cystoides intestinalis (PCI) is characterized by gas-filled cysts formed within submucosa and/or subserosa of the intestine.^1^ Pneumatosis cystoides intestinalis is more frequently found in the colon and small intestine. Lesions with colonic localizations are called colonic PCI, with predominant submucosa involvement.^2^ Although it has been found PCI is in association with dysbacteriosis, inflammation, and physical injury of intestinal mucosa, the exact pathogenesis remains unknown.^3^ Pneumatosis cystoides intestinalis is easy to be misdiagnosed or underdiagnosed and generally found in colonoscopy or radiological imaging by accidence due to lack of specific symptoms. Currently, studies on colonic PCI are relatively absent and most of them are small sample size and lack long-term follow-up or prognosis evaluation. Hence, we respectively analyzed the clinical and endoscopic features of 192 PCI cases in order to improve the diagnosis and cognition of PCI. In addition, we sought to find potential factors associated with the lesion size and the prognosis of PCI.

## Materials and Methods

### Study Design and Participants

Data from a total of 192 patients diagnosed with colonic PCI at the PLA General Hospital Digestive Endoscopy Center from July 2015 to October 2021 were collected. Patients received colonoscopy (CF-H260AI, CF-HQ290I; Olympus, Tokyo, Japan) followed by standardized bowel preparation in accordance with current guidelines.^4^ Pneumatosis cystoides intestinalis is diagnosed with gas-filled cysts formed within submucosa and/or subserosa of the intestine.^1^ Each patient provided a signed informed consent form for operation including colonoscopy, biopsy, endoscopic therapy, and endoscopic ultrasonography (EUS). The study was approved by the Institutional Review Board of Chinese PLA General Hospital (approval number: S2021-638, date: January 20, 2022).

Clinical manifestations including gender, age, symptoms, concomitant diseases (history of polypectomy, respiratory diseases, diabetes, alpha-glucosidase inhibitor therapy, and chemotherapy/glucocorticoids), endoscopic features (lesion size and location), and combined diagnosis (polyps, colitis, melanosis coli, colorectal cancer, diverticulum, and ulcerative colitis) are retrospectively analyzed. Twenty-eight patients received EUS in this study, and features involving echo intensity, origination, and adjacent relationship of lesions were recorded. Six patients received computed tomography, and features including intraperitoneal free air, primary location, bowel dilatation, bowel wall thickening, ascites, and portomesenteric venous gas were evaluated. Outcomes were measured in 50 patients who underwent follow-up colonoscopy in our hospital.

As large cysts may compress the bowel lumen which leads to the occurrence of intestinal obstruction,^5^ we divided participants enrolled in our study into 2 groups based on cysts with a size of 2 cm. Clinical characteristics, endoscopic features, and outcomes were compared between patients with lesion size <2 cm (n = 101) and lesion size ≥2 cm (n = 91).

### Outcome Measurements

The primary outcomes were clinical and endoscopic features of PCI in 192 patients. Secondary outcomes were compared between patients with lesion size <2 cm and lesion size ≥2 cm on the features mentioned above. Factors associated with the prognosis of the disease were also evaluated in this study.

### Statistical Analysis

The statistical analysis was conducted with the Statistical Package for the Social Sciences 22.0 software (IBM Corp., Armonk, NY, USA). Categorical variables were presented as frequencies and proportions, compared by Chi-squared test or Fisher’s exact test. Continuous variables were presented as mean ± SD and compared by Student’s *t*-test or the Mann–Whitney *U*-test. A 2-tailed *P* <.05 was considered as statistically significance.

## Results

### Baseline Characteristics

A total of 192 patients with colonic PCI were retrospectively included. As shown in Table 1, 83 cases (43.23%) were females and 109 cases (56.77%) were males with a mean age of 54.82 ± 13.09 (range 15-84) years old. Most of the patients (82, 42.71%) were asymptomatic; the main presenting symptoms were abdominal pain (51, 26.56%) and diarrhea (18, 9.38%). In terms of the concomitant diseases, 40 patients (20.83%) had a history of polypectomy or abdominal surgery, 11 patients (5.73%) were with respiratory diseases, 16 patients (8.33%) had diabetes mellitus, and 9 cases (4.69%) of them received alpha-glucosidase inhibitor (α-GI) therapy. Besides, 6 patients (3.13%) had received chemotherapy or glucocorticoid therapy.

### Endoscopic Findings

**Figure 1.**
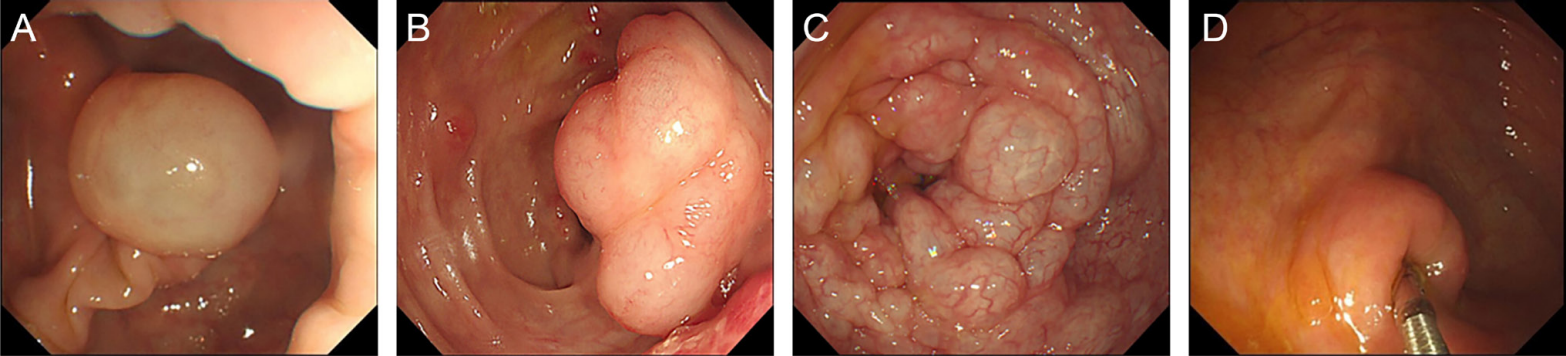
Endoscopic findings of colonic pneumatosis cystoides intestinalis. (A) Solitary, hemispherical, and translucent cyst in the colonic wall. (B) Cystic fusion of lesions. (C) Lesions with irregular beaded continuous distribution and smooth surface. (D) The lesions became depressed and deformed with compression of biopsy forceps.

Colonic PCI can be solitary (Figure 1A), fused (Figure 1B), or irregularly beaded forms (Figure 1C). Pneumatosis cystoides intestinalis lesions were solitary in 144 cases (75.00%) and multiple in 48 cases (25.00%). The lesions had smooth surfaces with slightly blue or colorless appearance, some of which may be accompanied by congested erosion of the mucosa. The lesions were soft when touched with the biopsy forceps and began to deform with compression (Figure 1D). The cysts became collapsed when biopsied followed by the release of the gas, without fluid outflowed.

The size of the lesions ranged from 0.5 to 5.0 cm, of which 91 cases (47.40%) were 2 cm or over. The lesions were more predominantly located in the right colon, the primary involved site was the ascending colon (54, 28.13%), followed by the ileocecal junction (53, 27.60%), transverse colon (40, 20.83%), hepatic flexure (11, 5.73%), descending colon (11, 5.73%), sigmoid colon (10, 5.21%), multiple bowel segments (10, 5.21%), and rectum (3, 0.51%). Patients may have other endoscopic diagnoses simultaneously, mainly consisting of colonic polyps (68, 35.42%), colitis (14, 7.29%), colorectal cancer (12, 6.25%), melanosis coli (6, 3.13%), colonic diverticulum (5, 2.60%), and ulcerative colitis (1, 0.52%) (Table 2).

The patients with lesion size <2 cm showed significant differences in symptoms, history of polypectomy/abdominal surgery, and history of α-GI compared to those with lesion size <2 cm (*P *< .05), while other factors such as gender, age, location, and concomitant diseases were not comparable (Tables 1 and 2).

### EUS Features

**Figure 2.**
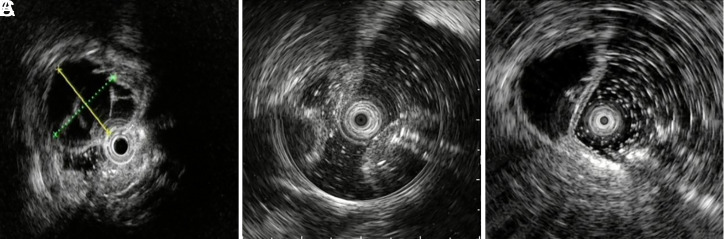
EUS features of colonic pneumatosis cystoides intestinalis. (A)–(C) The lesion was in the submucosa with peripheral enhancement, showing cystic or irregular hypoechoic changes, and hyperechoic areas inside.

A total of 28 patients (14.58%) underwent EUS. As shown in Figure 2A-C, the lesions showed irregular hypoechoic areas and were located in the submucosa with peripheral enhancement. Hyperechoic areas can be seen inside the lesion due to multiple linear or irregular gases, followed by acoustical shadows.

### Radiological Imaging Features

**Figure 3.**
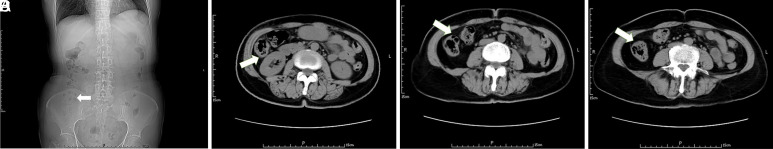
Radiological imaging features of colonic pneumatosis cystoides intestinalis. (A) Abdominal plain film showed multiple circular light transmission areas with scattered or beaded distribution (as shown by the arrow). (B–D) Small cystic and clustered low-density shadows under the serosa of ascending colon and hepatic flexure (as shown by the arrow).

A total of 6 patients received computed tomography (CT) examination. The abdominal plain film showed multiple circular or semicircle light transmission areas with different sizes and scattered or beaded distribution (Figure 3A). Small cystic and clustered low-density shadows can be seen under the serosa of ascending colon and hepatic flexure (Figure 3B-D). Free air, bowel dilatation, bowel wall thickening, ascites, and portomesenteric venous gas were not seen in these patients.

### Pathological Diagnosis

A total of 26 patients (13.54%) underwent endoscopic biopsy. The pathological result of biopsy was mainly chronic mucosal inflammation. Lymphoid hyperplasia in lamina propria was observed in part of the patients, with adenomatous hyperplasia of local glands. One patient showed abnormal and dilated vessels under the mucosa.

### Treatments and Prognosis

**Figure 4.**
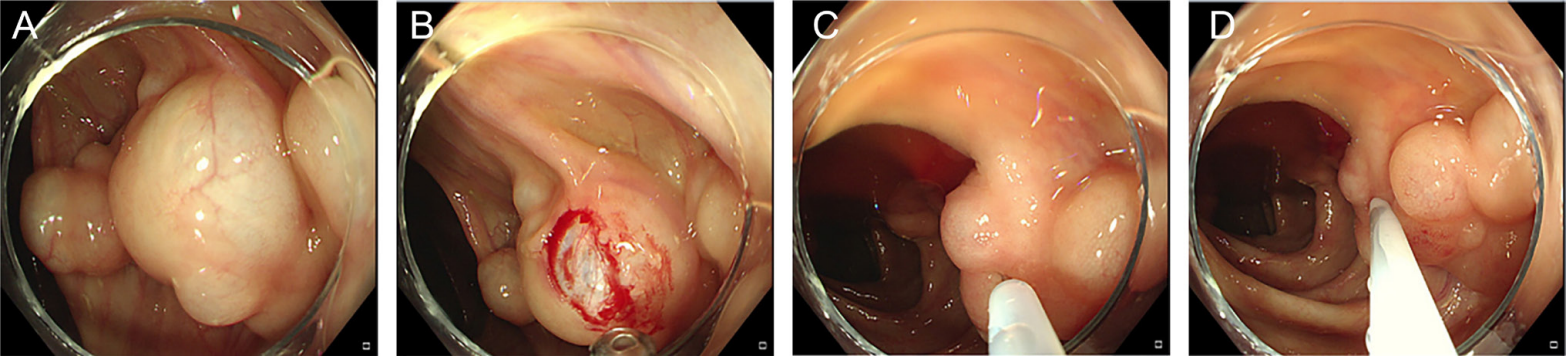
The lesion was soft when touched with biopsy forceps, and the transparent cyst wall can be seen after biopsy. The cyst became collapsed with the aspiration of a 23G injection needle.

The lesions of 16 patients disappeared completely after biopsy. One patient received endoscopic dissection. One patient was treated with a 23G injection needle (NM-400L-0423, Olympus, Tokyo, Japan), and the cyst became collapsed after aspiration of the gas (Figure 4A-D). In addition, a 70-year-old patient who suffered from diarrhea received hyperbaric oxygen therapy (HBOT) for 8 days and discontinued treatment due to otitis media. The lesions were alleviated significantly after 1-year follow-up. The rest of the patients did not receive any special treatments. A total of 50 patients (28.9%) underwent follow-up colonoscopy with a median follow-up period of 25 months (range 6-72 months). Among them, 28 cases (56.00%) disappeared spontaneously, and 22 patients (44.00%) remain unchanged. Age, gender, symptoms, concomitant diseases, solitary/multiple, location, and size, combined with polyps, were not associated with prognosis (Table 3).

## Discussion

Pneumatosis cystoides intestinalis was first discovered by Du Vernoi in autopsy specimens in 1730,^6^ then Hahn^7^ reported a surviving patient with PCI and proposed a classification in 1899. In a systematic analysis based on a Chinese database, Wu et al^8^ found the mean onset age of PCI was 45.3 ± 15.6 (range 2-81) years old with a male-to-female ratio of 2.4:1. In this study, the mean onset age was 54.82 ± 13.09 (range 15-84) years old with a male-to-female ratio of 1.3:1.

Pneumatosis cystoides intestinalis can be divided into primary type (15%) and secondary type (85%).^5^ Various factors have been reported to contribute to the formation of PCI: pyloric stenosis, chronic obstructive pulmonary disease (COPD), abdominal trauma or surgery, antibiotics, and malnutrition.^9,10^ The etiology of PCI can be explained by the following 3 hypotheses: (1) mechanical theory: increased intraluminal pressure caused by surgery, trauma, or colonoscopy resulted in mechanical damage of the intestinal wall and mucosal rupture, then the gas migrated from the gastrointestinal tract to the intestinal wall.^8^ A total of 40 patients (20.83%) in this study were found to have a history of polypectomy or abdominal surgery and their lesion size was more likely to be <2 cm (28.57% vs. 13.86%; *P *= .012). (2) Pulmonary theory: respiratory diseases involving COPD, interstitial pneumonia, and asthma may cause elevated alveolar pressure and rupture, leading to the occurrence of pneumomediastinum; then the gas is released into the intestinal wall along the aorta and mesenteric vessels.^11^ In this study, 11 patients (5.73%) were accompanied by chronic pulmonary diseases such as asthma and pulmonary bullae. (3) Bacterial theory: overgrown gas-producing bacteria penetrate the intestinal mucosal barrier and ferment in the wall, causing the formation of cysts.^12^

Recently, some reports found that PCI was related to α-GI therapy including acarbose and voglibose.^13,14^ Those drugs inhibit the absorption of carbohydrates. Accumulated carbohydrate was fermented by intestinal bacteria, producing a volume of gas.^15^ Besides, hyperglycemic status may decrease the intestinal peristalsis, causing elevated pressure within the lumen. Cysts are formed with the entrance of gas-producing bacteria into the submucosa of the intestinal wall.^16^ In this study, 16 patients (17.58%) were complicated with diabetes, among which 9 patients (4.69%) received α-GI therapy and the lesion size was more likely to be less than 2 cm (7.92% vs. 1.10; *P *= .037). Unlike patients with a history of polypectomy or abdominal surgery, no defects occurred in the intestinal mucosa for patients with α-GI therapy. This may explain why lesions in patients who received α-GI therapy were smaller while for those who had polypectomy or abdominal surgery were larger. A retrospective study showed that the incidence of bowel ischemia was significantly lower in patients with α-GI therapy.^17^ Besides, PCI achieved remission with relative ease after ceasing the α-GI therapy.^13^ Our conclusion combined with these findings indicated that PCI caused by α-GI was less likely to be a high-risk condition. It has also been reported that chemotherapy may be associated with PCI.^18^ Chemotherapy increases the risk of intestinal infection and leads to overgrowth of gas-producing bacteria. Moreover, chemotherapy may cause severe diarrhea and induce intestinal mucosa defection.^19^ Four patients had received chemotherapy in this study, which may contribute to PCI. In conclusion, PCI should be considered when patients combined with the above conditions show gastrointestinal symptoms.

Patients with PCI may be asymptomatic or present with non-specific symptoms such as abdominal pain, diarrhea, abdominal distension, constipation, bloody stool, loss of appetite, and weight loss.^11^ A total of 82 patients (42.71%) had no obvious symptoms in our study. Our research showed patients with lesion size <2 cm and lesion size ≥2 cm have a significant difference in clinical manifestations (*P *= .026), while no significant difference was observed in gender, age, concomitant diseases, location, and multiple lesions.

Colonoscopy is the main diagnostic method for colonic PCI. Under endoscopy, PCI can be presented as vacuolated, botryoid or beaded, linear or cobblestone pattern, and irregular. Vacuolated lesions tend to be primary and should be differentiated from polyps, botryoid or beaded lesions should be differentiated from intestinal tuberculosis, while irregular lesions should be differentiated from Crohn’s disease and tumors.^3^

Pneumatosis cystoides intestinalis preferentially involved colon (61.8%), with the right colon being the most common site in secondary type.^20,21^ Our study showed lesions were mainly located in the ascending colon (54, 28.13%) and ileocecal junction (53, 27.60%) respectively, followed by transverse colon (40, 20.83%), which was basically consistent with other literatures. This finding may be due to anatomic difference between the left and right colon as the intestinal wall is thinner in the right colon. Besides, phenotypes of the bacterium are markedly different on the 2 sides of the colon, which may contribute to the formation of gas.^22^ EUS findings of cystic or irregular hypoechoic changes with multiple linear or irregular hyperechoic areas are helpful to distinguish PCI from other diseases. Pathological diagnosis of PCI was mostly non-specific inflammation.^23^ Results of pathological biopsy in this study were mostly found to be chronic mucosal inflammation.

Radiological imaging also plays an essential part for the diagnosis of PCI. Cysts usually appear as honeycomb signs or grape-shaped clusters along the intestinal wall under radiological imaging.^24^ It had been proved that CT findings including portomesenteric venous gas, thickened bowel wall, dilated bowel lumen, and ascites were significantly associated with increased mortality of pneumatosis.^25^ Intraperitoneal free air can be observed in about 2% PCI lesions in the colon and 15% in the small intestine, and it has to be differentiated carefully with acute abdomen.^26^ Only 6 patients received CT examination and none of them showed IFA in our study.

Currently, no consensus on managements of PCI has been made. Treatments that can be adopted are as follows: (1) Observation: patients without pronounced symptoms can receive regular follow-up without any interventions. (2) Conservation therapy: patients with mild symptoms can be managed by antibiotics in combination with an elemental diet. Antibiotics including metronidazole and quinolone can target intestinal bacteria and reduce hydrogen production.^5,27^ (3) Oxygen therapy or HBOT could be considered for patients with obvious symptoms but no surgical indications.^3^ A patient received HBOT in our study and a follow-up colonoscopy showed lesions became effectively alleviated; (4) endoscopic therapy: the gas inside the cyst can be removed through puncture by fine needle aspiration.^3,28^ In this study, 1 patient received injection needle aspiration, and the lesions disappeared after aspiration, and 1 patient received endoscopic dissection. (5) Surgery: early surgery invention is reserved for patients with signs of complications including intestinal perforation, intestinal obstruction, intestinal ischemia, and necrosis.^29^ For 50 patients who underwent follow-up colonoscopy in our study, 28 cases (56.00%) disappeared spontaneously, while 22 cases (44.00%) persisted. The prognosis was found to have no relationship with the lesion size; this may be due to the relatively small number of patients received follow-up colonoscopy in this study. A larger cyst has a risk for compressing the intestinal lumen which may lead to severe complications such as intestinal obstruction and volvulus,^30^ while most lesions were found occasionally in screening colonoscopy and the majority of patients were asymptomatic without any severe complications in our study. Besides, the prognosis of PCI in our study refers to whether the lesion will disappear spontaneously or remain unchanged and does not evaluate risks for complications. Therefore, recognizing features for larger lesions is still considerable in clinical practice.

This study has some limitations. First, the study was based on clinical records in a retrospective manner. Second, factors including gender, age, clinical manifestations or concomitant diseases, lesion size, location, and single/multiple lesions were not found to be related to the outcome of PCI in this study. The reason might be limited number of patients receiving follow-up colonoscopy and short follow-up period.

In conclusion, the etiology of PCI is still not clear and may be primary or secondary to some systemic diseases. Due to the lack of specific clinical manifestations, diagnosis of PCI is mainly dependent on colonoscopy and EUS, and it is easy to be confused with polyps, cancer, or inflammatory bowel disease. Awareness of PCI should be improved for endoscopic operators in order to avoid unnecessary medical procedures and reduce the psychological and economic burden of patients. In addition, further researches are needed toward the etiology of PCI, the efficacy of different therapeutic strategies, and the outcome of the disease.

## Figures and Tables

**Table 1.** Clinical Characteristics of 192 Patients with Colonic Pneumatosis Cystoides Intestinalis

Characteristics	Lesion size <2 cm (n = 101)	Lesion size ≥2 cm (n = 91)	Total (n = 192)	*P*
Age (mean ± SD, years)	55.71 ± 13.98	53.82 ± 12.02	54.82 ± 13.09	.319
Gender (male/female)	62/39	47/44	109/83	.174
Symptoms, n (%)				.026
Absent	47 (46.53)	35 (38.46)	82 (42.71)	
Abdominal pain	24 (23.76)	27 (29.67)	51 (26.56)	
Constipation	10 (9.90)	4 (4.40)	14 (6.77)	
Diarrhea	4 (3.96)	14 (15.38)	18 (9.38)	
Bloody stool	8 (7.92)	4 (4.40)	12 (6.25)	
Distention	4 (3.96)	6 (6.59)	10 (5.21)	
Elevated tumor markers^a^	3 (2.97)	0 (0.00)	3 (1.56)	
Weight loss	1 (0.99)	1 (1.10)	2 (1.04)	
Concomitant diseases, n (%)				
Prior polypectomy/surgery^b^	14 (13.86)	26 (28.57)	40 (20.83)	.012
Respiratory diseases^c^	8 (7.92)	3 (3.30)	11 (5.73)	.169
Diabetes	12 (11.88)	4 (4.40)	16 (17.58)	.061
History of α-GI therapy	8 (7.92)	1 (1.10)	9 (4.69)	.037
Chemotherapy/glucocorticoids^d^	4 (3.96)	2 (2.20)	6 (3.13)	.775

^a^Tumor marker: CEA, CA19-9.

^b^Abdominal surgery: colorectal surgery, appendicectomy, cholecystectomy, and ovariectomy.

^c^Respiratory diseases: asthma, infection, cancer, bullae, calcification, and tuberculosis.

^d^Four patients received chemotherapy including XELOX (capecitabine plus oxaliplatin) and ELF (5-fluorouracil, leucovorin, and etoposide). Two patients received glucocorticoid therapy for membranous nephropathy and asthma, respectively.

α-GI, alpha-glucosidase inhibitor.

**Table 2.** Endoscopic Findings of 192 Patients with Colonic Pneumatosis Cystoides Intestinalis

Endoscopic Findings	Lesion size <2 cm (n = 101)	Lesion size ≥2 cm (n = 91)	Total	*P*
Solitary/multiple	75/26	69/22	144/48	.802
Lesion location, n (%)				
Ileocecal junction	29 (28.71)	24 (26.37)	53 (27.60)	.226
Ascending colon	23 (22.77)	31 (34.07)	54 (28.13)	
Hepatic flexure	3 (2.97)	8 (8.79)	11 (5.73)	
Transverse colon	24 (23.76)	16 (17.58)	40 (20.83)	
Descending colon	7 (6.93)	4 (4.40)	11 (5.73)	
Sigmoid colon and rectum	9 (8.91)	4 (4.40)	13 (6.77)	
Multiple bowel segments	6 (5.94)	4 (4.40)	10 (5.21)	
Combined diagnosis, n (%)				
Colonic polyps	38 (37.62)	30 (32.98)	68 (35.42)	.501
Colitis	9 (8.91)	5 (5.49)	14 (7.29)	.363
Melanosis coli	3 (2.97)	3 (3.30)	6 (3.13)	1.000
Colorectal cancer	4 (3.96)	8 (8.79)	12 (6.25)	.167
Colonic diverticulum	3 (2.97)	2 (2.20)	5 (2.60)	1.000
Ulcerative colitis	1 (0.99)	0 (0.00)	1 (0.52)	1.000

**Table 3.** The Prognosis of 50 Patients who Received a Follow-up Colonoscopy

	Disappeared spontaneously (n = 28)	Remained unchanged (n = 22)	*P*
Age (mean ± SD, years)	54.79 ± 12.54	55.95 ± 10.51	.727
Gender (male/female)	17/11	14/8	.833
Symptomatic, n (%)	14 (50.00%)	13 (40.91)	.522
Concomitant diseases, n (%)			
Present	19 (67.86%)	11 (50.00%)	.201
Absent	9 (32.14%)	11 (50.00%)	
Solitary/multiple	21/7	17/5	.852
Lesion location			
Ileocecal junction	8 (28.57%)	6 (27.27%)	.469
Ascending colon	10 (35.71%)	8 (36.36%)	
Hepatic flexure	1 (3.57%)	0 (0.00%)	
Transverse colon	5 (17.56%)	6 (27.27%)	
Descending colon	4 (14.29%)	1 (4.55%)	
Sigmoid colon/rectum	0 (0.00%)	1 (4.55%)	
Lesion size			
<2 cm	14 (50.00%)	14 (63.64%)	.335
≥2 cm	14 (50.00%)	8 (36.36%)	
Combined with polyps, n (%)	10 (35.71%)	9 (40.91%)	.707
